# Prediction of Prognosis in Adult Patients With Carbapenem-Resistant *Klebsiella pneumoniae* Infection

**DOI:** 10.3389/fcimb.2021.818308

**Published:** 2022-01-11

**Authors:** Jihui Chen, Yu Yang, Huimin Yao, Shuhong Bu, Lixia Li, Fang Wang, Feng Chen, Huijuan Yao

**Affiliations:** ^1^ Department of Pharmacy, Xinhua Hospital, School of Medicine, Shanghai Jiao Tong University, Shanghai, China; ^2^ School of Pharmacy and Medicine, Tonghua Normal University, Jilin, China; ^3^ Clinical Laboratory, Xinhua Hospital, School of Medicine, Shanghai Jiao Tong University, Shanghai, China

**Keywords:** carbapenem-resistant, *Klebsiella pneumoniae*, infection, nomogram, mortality

## Abstract

**Objective:**

Carbapenem-resistant *Klebsiella pneumoniae* (CRKP) infections are associated with poor patient outcomes. We aimed to analyze the clinical information of adult patients with CRKP infection in order to establish a nomogram for mortality risk as well as to determine the treatment effectiveness of different antimicrobial regimens.

**Methods:**

Adult patients diagnosed with CRKP infection in a tertiary hospital in Shanghai between September 2019 and March 2021 were included. The clinical characteristics and clinical outcomes of these patients were analyzed.

**Results:**

A total of 199 cases of CRKP infection were examined. Five factors, namely age ≥65 years, respiratory failure, Sequential Organ Failure Assessment score, serum procalcitonin ≥5 ng/mL, and appropriate treatments in 3 days, were found to be associated with 30-day mortality. Upon incorporating these factors, the nomogram achieved good concordance indexes of 0.85 (95% confidence interval [CI]: 0.80–0.90) and well-fitted calibration curves. Receiver-operating characteristic curves for 7-, 15-, and 30-day survival had areas under the curve of 0.90, 0.87, and 0.88, respectively. Three-drug combination therapy was observed to be associated with lower mortality in the high-risk group (adjusted hazard ratio = 0.24, 95% CI: 0.06–0.99) but not in the low-risk group. Ceftazidime–avibactam, fosfomycin, and amikacin were effective against infections caused by CRKP. Tigecycline improved the treatment efficiency in 7 days, but a trend toward increased mortality was seen (HR, 1.69; 95% CI: 0.98–2.94; *P* = 0.061).

**Conclusion:**

The antimicrobial regimen efficacy data and the predictive nomogram established in this study can help clinicians in identifying high-risk adult patients with CRKP infection, improving the therapeutic effect, and reducing mortality.

## Introduction

*Klebsiella pneumoniae* (KP) is one of the most common pathogens associated with nosocomial infections such as pneumonia, urinary tract infections, bloodstream infections, and sepsis (Bengoechea and Sa Pessoa, 2019; Rodríguez-Medina et al., 2019). Carbapenem-resistant KP (CRKP) infections are increasing globally (Munoz-Price et al., 2013; Brink, 2019), and in China, the detection rate of CRKP increased significantly from 9.2% in 2010 to 27.1% in 2021 (http://www.chinets.com/Data/GermYear).

CRKP infections have emerged as an important public health threat over the past decade in China owing to the limited treatment options and unsatisfactory outcomes (Durante-Mangoni et al., 2019; Tsioutis et al., 2021). Existing antibiotics such as tigecyclines, polymyxins, fosfomycin, and aminoglycosides are the currently available therapeutic options for CRKP infections, while ceftazidime–avibactam is a newly developed antibiotic (Tilahun et al., 2021). Moreover, studies have indicated that combination therapies are often more effective than monotherapies (Pournaras et al., 2011; Tumbarello et al., 2012; Falcone et al., 2016), but their use must be balanced against possible disadvantages such as superinfection, increased incidence of adverse effects, and high cost (Agyeman et al., 2020a).

Therefore, a comprehensive understanding of the clinical features, risk factors, and outcomes of patients with CRKP infection are important. Also, there is an urgent need to establish a scoring system for predicting mortality to assess the severity of the illness and the risk of mortality.

In the present retrospective cohort study, we analyzed the clinical information of adult patients with CRKP infection. Based on the Cox proportional regression, we established a practical and operable nomogram scoring system to help clinicians evaluate the prognosis. We also examined the impact of different antimicrobial regimens on the prognosis to enhance therapeutic efficiency.

## Patients and Methods

### Study Design

The present retrospective study was conducted in a 2600-bed tertiary-care hospital in Shanghai, China from September 2019 to March 2021. Patients ≥18 years who were infected with CRKP were included in this study. Patients with CRKP colonization (with positive CRKP cultures but without any clinical signs of infection), on an end-of-life care pathway, or those who were discharged within 2 days of detection of CRKP were excluded from the study. The study protocol has been approved by the Ethics Committee of XinHua Hospital Affiliated with Shanghai Jiao Tong University School of Medicine (XHEC-D-2021-130). The provision of informed consent from the study participants was waived off.

### Data Collection

We reviewed and extracted data from the patients’ electronic medical records and charts into the data collection form. Patient demographics, baseline diseases, comorbidities, and clinical and microbiological data of the infection, as well as therapeutic procedures, are undertaken, were collected for analyses.

### Definitions

The baseline diseases, comorbidities, and clinical status of the infection in the study participants were evaluated on the day of CRKP detection. Respiratory failure was defined as a PaO_2_/FiO_2_ <200 while breathing spontaneously and/or the need for mechanical ventilation. Immunosuppression referred to the receipt of corticosteroids (≥10 mg or an equivalent daily dosage) for >2 weeks or antineoplastic chemotherapy within the past 4 weeks. Appropriate therapy in 3 days referred to the therapy with at least one drug to which the CRKP was susceptible *in vitro* and initiated before or within 72 h after the antibiogram results were reported to the clinicians and continued for at least 48 h to allow the therapeutic agents to take effect. The number of anti-KP drugs referred to the number of antibiotics intended to be used against CRKP, which were administered at the same time within 7 days after CRKP detection. All laboratory variables from the blood were obtained on the day of CRKP detection (the procalcitonin results of the next day were also included).

### Antimicrobial Susceptibility Testing

All CRKP isolates were identified by matrix-assisted laser desorption ionization-time of flight mass spectrometry (MALDI-TOF MS; Bruker Daltonics, Germany), while the minimum inhibitory concentrations (MICs) of antibiotics were determined using the Vitek 2 system and the AST-GN card (bioMérieux, France). The Kirby–Bauer disk diffusion method was employed as a supplementary susceptibility test. Carbapenem resistance of CRKP was defined as a minimum inhibitory concentration of ≥4 μg/mL for meropenem/imipenem or ≥2 μg/mL for ertapenem with reference to the CLSI criteria (Clinical and Laboratory Standards Institute, 2021).

### Outcome Measurements

The primary outcome was the time to all-cause death up to 30 days from CRKP detection. The secondary outcome was the effect of treatment on CRKP infection, which was defined as effective (cure or improvement in the infection status) or noneffective (stable or deterioration of infection) within 7 and 15 days, based on the clinical, radiological, and laboratory findings. The cost incurred in administering the antimicrobial agents was also analyzed in the 30-day surviving patients.

### Statistical Analyses

All analyses were performed using R version 4.1.0 (The R Foundation, Vienna, Austria). Categorical variables were presented as numbers and percentages, whereas the continuous variables were presented as mean ± standard deviation or medians (interquartile range [IQR]). Categorical variables were tested with the Chi-squared test or Fisher’s exact test, as deemed appropriate. Continuous variables with a normal distribution were compared using Student’s *t*-test or Mann–Whitney U-test. *P* < 0.05 was considered to indicate statistical significance, and all tests performed were two-tailed.

Risk factors were identified by univariable and multivariable Cox regression analyses for predicting mortality. The best subset selection based on the minimal Bayesian information criterion (BIC) was performed using the leaps package in R. Hazard ratio (HR) and 95% confidence intervals (CIs) were calculated to evaluate the strength of any association, and Kaplan–Meier (KM) survival curves were generated with log-rank test analysis. The nomogram was constructed using the rms package in R software. All patients were classified into either high-risk or low-risk groups based on the cut-off point obtained using the survminer package in R software.

The predictive performance of the nomogram was evaluated based on the discriminating ability and calibration results (Steyerberg and Vergouwe, 2014). Discrimination was assessed through the concordance index (C-index) and time-dependent receiver operating characteristic (ROC) curves using the timeROC package in R software. The model was validated using bootstrapped resampling, and the calibration curves for 7, 15, and 30 days were applied using 1000 repeated samples to evaluate the consistency of model prediction. The clinical usefulness was examined based on the net benefit by decision curve analysis (DCA) using the ggDCA package in R software.

In order to evaluate the clinical effect of antimicrobial agents in 7, 15 days, multivariate logistic regression analysis was applied to estimate the adjusted odds ratio (aOR) and 95% CI. Univariate analyses were performed to determine the predictors of the high treatment cost of antimicrobial agents or all medications during hospitalization. Factors showing correlations (*P* < 0.1) with a high cost, after evaluating for collinearity, were further analyzed by multiple logistic regression using a backward stepwise selection with the Akaike information criterion (AIC).

## Results

### Characteristics of Patients With CRKP

From September 2019 to March 2021, a total of 199 adult patients identified with CRKP infection were enrolled in this study. There were 191 patients for whom 30-day survival data were available; their median age was 71 years (IQR 61–81 years), and 142 (74.3%) of them were men. The demographic details, clinical characteristics, and treatment regimens are shown in **Table 1**. The 7-, 15-, and 30-day mortality rates were 13.6% (26/191), 22.0% (42/191), and 26.7% (51/191), respectively.

**Table 1:** Characteristics of patients with carbapenem-resistant *Klebsiella pneumoniae* infections.

Characteristic	All patients n = 191	30-day non-survivors n = 51 (26.7%)	30-day survivors n = 140 (73.3%)	*P* value
**Patient variables**				
Male sex, n (%)	142 (74.3)	37 (72.5)	105 (75.0)	0.732
Age, y, median (IQR)	71 (61, 81)	76 (65, 85)	68 (60.75, 78)	0.001
**Baseline disease or comorbidity, n (%)**				
Diabetes mellitus	58 (30.4)	14 (27.5)	44 (31.4)	0.597
Cardiovascular disease	122 (63.9)	30 (58.8)	92 (65.7)	0.381
Cerebrovascular disease	98 (51.3)	19 (37.3)	79 (56.4)	0.020
Renal disease	47 (24.6)	17 (33.3)	30 (21.4)	0.094
Hematological Disease	14 (7.3)	2 (3.9)	12 (8.6)	0.360
Digestive diseases	58 (30.4)	18 (35.3)	40 (28.6)	0.372
Malignant solid tumor	34 (17.8)	12 (23.5)	22 (15.7)	0.215
Surgery within the previous 30 days	131 (68.6)	33 (64.7)	98 (70.0)	0.486
Immunosuppressant use	169 (88.5)	47 (92.2)	122 (87.1)	0.342
ICU	162 (84.8)	44 (86.3)	118 (84.3)	0.735
**Clinical status, n (%)**				
Respiratory failure	49 (25.7)	24 (47.1)	25 (17.9)	<0.001
Heart failure	44 (23.0)	16 (31.4)	28 (20.0)	0.101
MODS	16 (8.4)	12 (23.5)	4 (2.9)	<0.001
SOFA score, median (IQR)	3 (3, 5.5)	5 (3, 8)	3 (2, 5)	<0.001
**Invasive procedure and/or devices, n (%)**				
Central venous catheterization	181 (94.8)	51 (100)	130 (92.9)	0.989
Urinary catheterization	167 (87.4)	47 (92.2)	120 (85.7)	0.242
Gastric catheterization	165 (86.4)	45 (88.2)	120 (85.7)	0.654
CRRT	18 (9.4)	9 (17.6)	9 (6.4)	0.024
**Type of infections, n (%)**				
Catheter-related infection	7 (3.7)	1 (2.0)	6 (4.3)	0.677
Pneumonia	120 (62.8)	35 (68.6)	85 (60.7)	0.318
Intra-abdominal infection	15 (7.9)	3 (5.9)	12 (8.6)	0.763
Urinary tract infection	27 (14.1)	6 (11.8)	21 (15.0)	0.571
Gastrointestinal infection	3 (1.6)	1 (2.0)	2 (1.4)	1
Primary bloodstream infection	18 (9.4)	5 (9.8)	13 (9.3)	1
Skin and soft-tissue infection	4 (2.1)	0 (0)	4 (2.9)	1
CNS infection	1 (0.5)	0 (0)	1 (0.7)	1
**Laboratory variables from blood, Mean ± SD**				
WBC, × 10^9^/L	11.20 ± 5.78	12.61 ± 6.46	10.68 ± 5.44	0.045
ANC, × 10^9^/L	9.35 ± 5.22	10.63 ± 5.56	8.88 ± 5.02	0.044
Lymphocyte, × 10^9^/L	9.46 ± 6.95	7.71 ± 6.2	10.10 ± 7.11	0.040
Hemoglobin, g/L	95 ± 21	90 ± 20	97 ± 22	0.046
Platelet, × 10^9^/L	216 ± 134	162 ± 122	235 ± 134	0.001
CRP, mg/L	85 ± 59	101 ± 58	79 ± 58	0.028
PCT, ng/mL	2.70 ± 8.91	6.89 ± 16.31	1.22 ± 2.62	0.001
Albumin, g/L	32.3 ± 5.5	30.1 ± 6.3	33.1 ± 4.9	0.001
Antimicrobial regimens^a^, n (%)				
Carbapenems	92 (48.2)	28 (54.9)	64 (45.7)	0.262
BL-BLI^b^	63 (33.0)	20 (39.2)	43 (30.7)	0.270
Broad spectrum β-lactams	12 (6.3)	0 (0)	12 (8.6)	0.038
Ceftazidime-avibactam	12 (6.3)	0 (0)	12 (8.6)	0.038
Tigecycline	69 (36.1)	24 (47.1)	45 (32.1)	0.059
Polymyxin B	12 (6.3)	3 (5.9)	9 (6.4)	1
Aminoglycosides	36 (18.8)	4 (7.8)	32 (22.9)	0.021
Fosfomycin	47 (24.6)	6 (11.8)	41 (29.3)	0.013
Fluoroquinolones	13 (6.8)	2 (3.9)	11 (7.9)	0.519
Others^c^	32 (16.8)	6 (11.8)	26 (18.6)	0.269
Number of anti-KP drugs, n (%)				0.022
1	47 (24.6)	17 (33.3)	30 (21.4)	
2	93 (48.7)	26 (51.0)	67 (47.9)	
3	51 (26.7)	8 (15.7)	43 (30.7)	
Appropriate treatments in 3 days, n (%)	116 (60.7)	23 (45.1)	93 (66.4)	0.008
Outcomes of infection^d^				
Effective in 7 days, n (%)	78 (41.1)	12 (23.5)	66 (47.5)	0.004
Effective in 15 days, n (%)	127 (66.5)	15 (29.4)	112 (80.6)	<0.001
Cost of antimicrobial agents^e^, USD	7697 ± 10516	5648 ± 6830	8459 ± 11521	0.112
Cost of medications^e^, USD	16297 ± 17920	15048 ± 14072	16762 ± 19181	0.560

a Antimicrobial regimens referred to the antibiotics intended to be used against CRKP administered at the same time within 7 days after CRKP was detected.

b β-lactam/β-lactamase inhibitors (BL-BLI) included cefoperazone-sulbactam and piperacillin-tazobactam.

c Other drugs included tetracyclines and sulfamethoxazole-trimethoprim.

d The outcomes of infection were defined as effective (cure or improvement) or noneffective treatment (stable or deterioration) according to clinical, radiological, and laboratory findings.

e USD1 = CNY6.5 in year 2021.

IQR, interquartile range; ICU, intensive care unit; MODS, multiple organ dysfunction syndrome; SOFA, Sequential Organ Failure Assessment; CRRT, continuous renal replacement therapy; CNS, central nervous system; WBC, white blood count; ANC, absolute neutrophil count; CRP, c-reactive protein; PCT, procalcitonin; KP, Klebsiella pneumonia.

### The Mortality Risk Prediction Model and Nomogram

**Figure 1:**
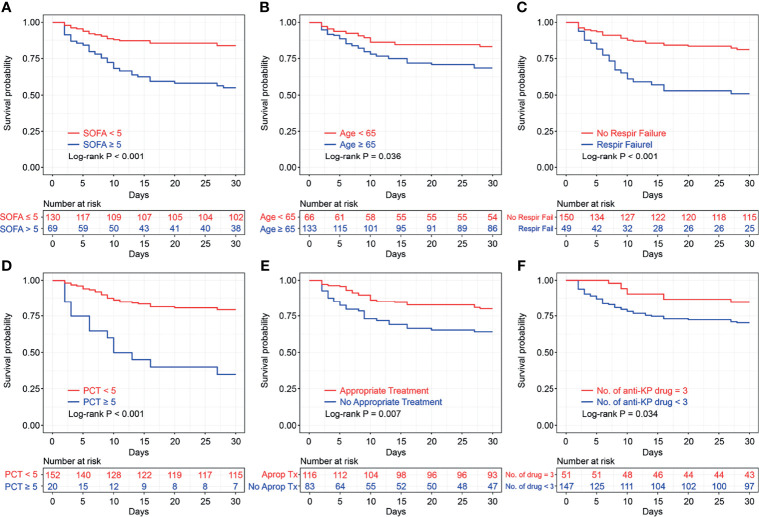
Kaplan–Meier survival plots of Sequential Organ Failure Assessment score **(A)**, age **(B)**, respiratory failure **(C)**, procalcitonin **(D)**, appropriate treatments in 3 days **(E)**, and number of anti-KP drugs **(F)**, with log-rank test. SOFA, Sequential Organ Failure Assessment; PCT, procalcitonin KP, *Klebsiella pneumoniae*.

**Figure 2:**
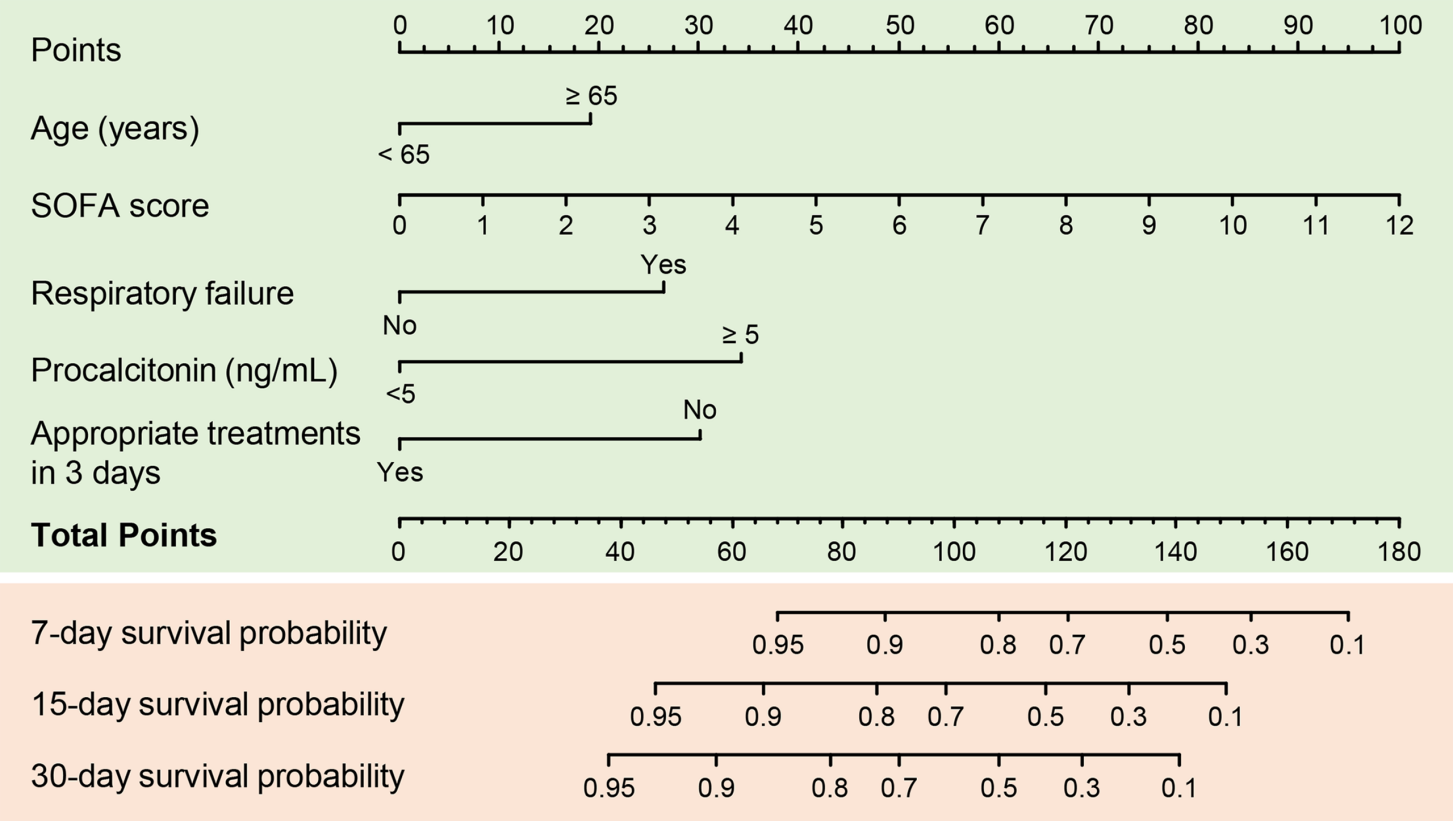
A predictive nomogram for predicting 7-, 15-, and 30-day mortality in patients with carbapenem-resistant *K. pneumoniae* infection. SOFA, Sequential Organ Failure Assessment.

Variables with *P* values < 0.1 in univariate Cox regression analyses and other important clinical variables were included in the multivariate analysis (**Table 2**). Multivariate Cox analysis identified five variables for the final model. The KM curves were plotted for the five variables (**Figure 1**). Among them, presence of respiratory failure (HR, 2.67; 95% CI: 1.44–4.97; *P* = 0.002), higher Sequential Organ Failure Assessment score (HR per one-point increment, 1.36; 95% CI: 1.22–1.51; *P* < 0.001), procalcitonin (PCT) > 5 ng/mL (HR, 3.54; 95% CI: 1.82–6.86; *P* < 0.001), and appropriate treatments in 3 days (HR, 0.33; 95% CI, 0.17–0.63; *P* < 0.001) were the independent factors for fatal outcomes. The above independent factors, along with age > 65 years (HR, 2.03; 95% CI: 0.83–4.98; *P* < 0.121), were used to establish the nomograms to predict the mortality in the patients with CRKP (**Figure 2**).

**Table 2:** Univariate and multivariate Cox regression analysis of risk factors for 30-day mortality.

Variable	Univariate analysis			Multivariate analysis		
	HR^a^	95%CI for HR	*P* value	HR	95%CI for HR	*P* value
Sex, male vs female	0.88	0.48-1.64	0.700			
Age, y, ≥65 vs <65	2.01	1.03-3.92	0.040	2.03	0.83-4.98	0.121
Respiratory failure	3.24	1.87-5.63	<0.001	2.67	1.44-4.97	0.002
Heart failure	1.57	0.87-2.84	0.133			
MODS	5.54	2.89-10.63	<0.001			
SOFA, 1-point increments	1.28	1.17-1.40	<0.001	1.36	1.22-1.51	<0.001
WBC, × 10^9^/L						
4-10 vs <4	0.29	0.10-0.82	0.020			
≥10 vs <4	0.64	0.25-1.65	0.358			
Hemoglobin, g/L, <80 vs ≥80	1.96	1.13-3.41	0.017			
Platelet, × 10^9^/L, <100 vs ≥100	3.11	1.76-5.49	<0.001			
CRP, mg/L, ≥150 vs <150	1.88	1.04-3.39	0.036			
PCT, ng/mL, ≥5 vs <5	4.59	2.39-8.83	<0.001	3.54	1.82-6.86	<0.001
Albumin, g/L, <30 vs ≥30	4.1	2.30-7.31	<0.001			
Tigecycline	1.69	0.98-2.94	0.061			
Aminoglycosides	0.32	0.12-0.89	0.029			
Fosfomycin	0.36	0.15-0.84	0.018			
Number of anti-KP drugs						
2 vs 1	0.76	0.41-1.41	0.389			
3 vs 1	0.38	0.16-0.88	0.024			
Appropriate treatments in 3 days	0.48	0.27-0.83	0.008	0.33	0.17-0.63	<0.001

a HRs were calculated comparing with comorbidity and without comorbidity.

CI, confidence interval; HR, hazard ratio; MODA, multiple organ dysfunction syndrome; SOFA, Sequential Organ Failure Assessment; WBC, white blood count; CRP, c-reactive protein; PCT, procalcitonin; KP, Klebsiella pneumonia.

**Figure 3:**
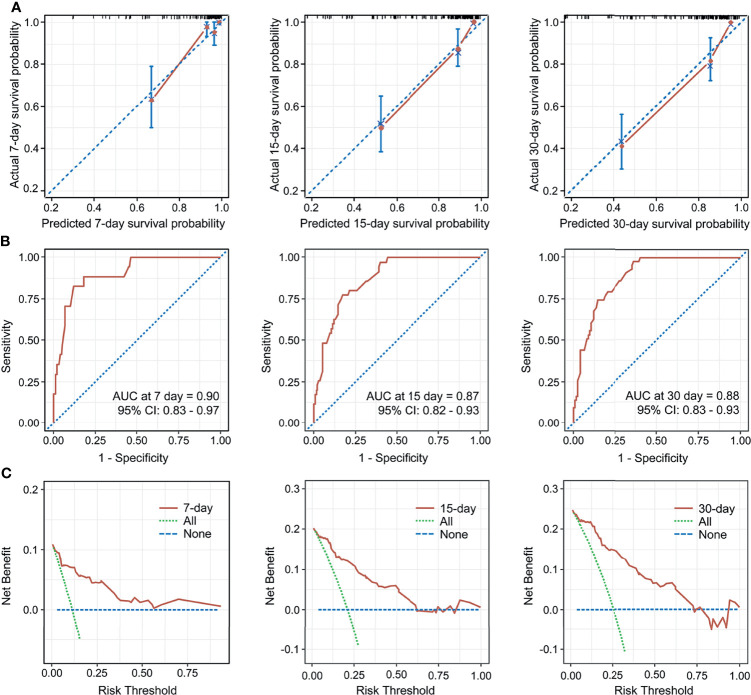
Calibration curves for the nomogram predicting 7-, 15-, and 30-day mortality **(A)**. AUC of time-dependent receiver operating characteristics (ROC) curves for predicting 7-, 15-, and 30-day survival **(B)**. Decision curve analysis for the 7-, 15-, 30-day survival nomogram **(C)**. CI, confidence interval.

The C-index for the established nomogram was 0.85 (95% CI: 0.80–0.90). Calibration plots and ROC curves were drawn to validate the predictive accuracy of the nomogram (**Figures 3A, B**). The 7-, 15-, and 30-day survival ROC results had areas under the curve of 0.90 (95% CI: 0.83–0.97), 0.87 (95% CI: 0.82–0.93), and 0.88 (95% CI: 0.83–0.93), respectively, which suggested a good concordance and a reliable ability. The decision curve demonstrated superior net benefit when the nomogram was used to compare the alternative approaches (**Figure 3C**).

**Figure 4:**
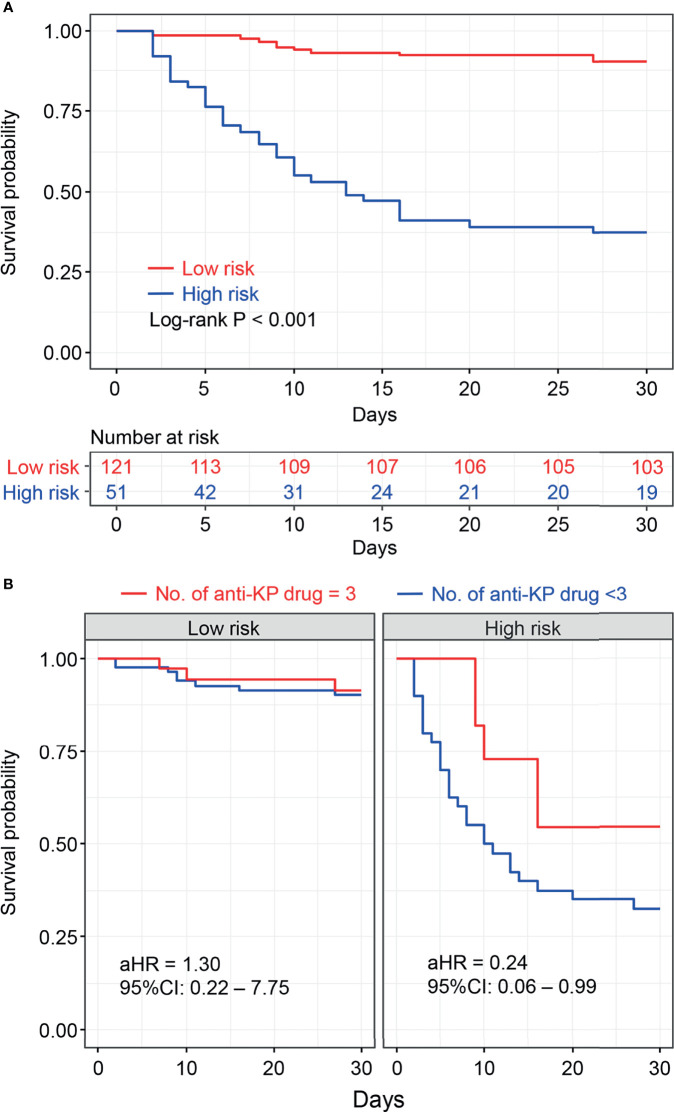
Kaplan–Meier survival plots of risk stratification according to the nomogram total points **(A)**. Kaplan–Meier survival plots for the number of anti- Klebsiella *pneumoniae* drugs associated with risk stratification were shown **(B)**, and adjusted hazard risk ratios (aHR) with 95% confidence intervals (CI) were estimated using the Cox regression analysis adjusted for age, sex, comorbidities, clinical status, and site of infection as potential confounders.

Based on the optional cut-off value of the nomogram points, risk stratification was done and the patients were divided into two risk groups, namely the low-risk group (total points <83) and high-risk group (total points ≥83). The KM survival curves revealed high discrimination between the two groups (**Figure 4A**).

### Antibiotic Therapy and Its Effectiveness

We continued to analyze the impact of different antimicrobial regimens on the prognosis. We found that 116 (60.7%) patients received appropriate antibiotic treatment within 72 h after CRKP infection diagnosis, which was an independent risk factor in the multivariate Cox regression analysis. The combination of drugs was also important for the treatment of CRKP infection. The KM survival analysis favored the three-drug combination therapy (*P* = 0.034, **Figure 1**).

Moreover, stratification analysis was performed according to the nomogram points. As shown in **Figure 4B**, the results indicated that the benefit of the three-drug combination therapy was more pronounced in the high-risk group [adjusted hazard ratio (aHR) = 0.24, 95% CI: 0.06–0.99] after adjusting for the confounding factors. A similar result was obtained in the stratification analysis based on SOFA scores (SOFA<5 and SOFA ≥5), as shown in **Supplementary Figure S1**. Among the 51 patients who received three-drug combination treatment, the most common combinations were tigecycline + fosfomycin + carbapenems and β-lactam/β-lactamase inhibitors (n = 9, n = 5, respectively).

**Figure 5:**
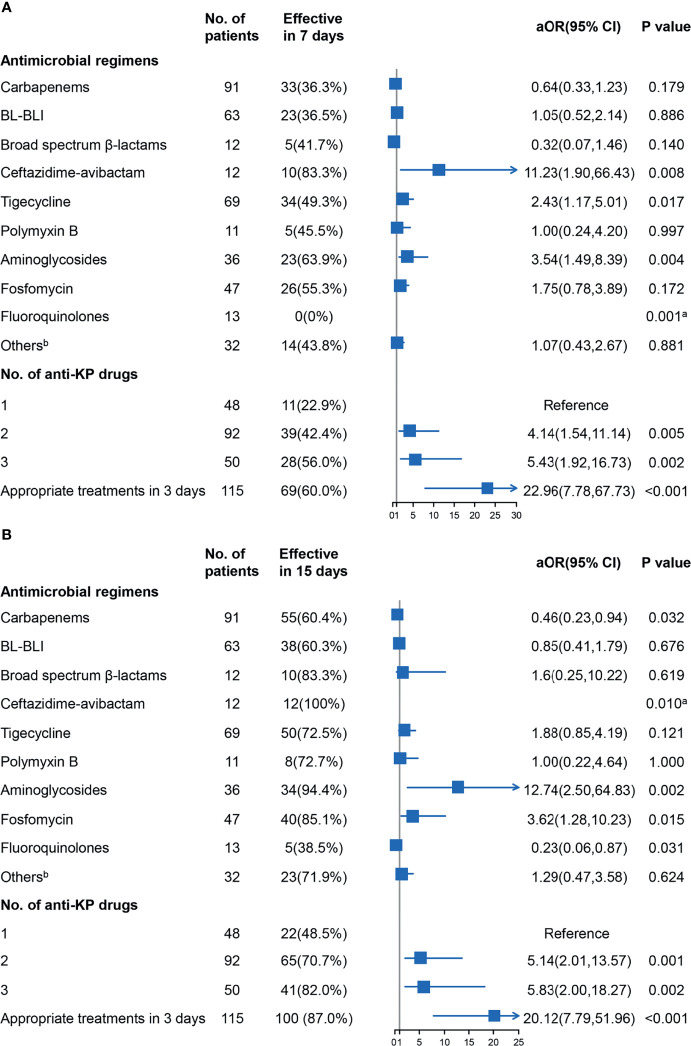
Results of logistic analysis of the associations between the antimicrobial agents and treatment effectiveness in 7 days **(A)** and 15 days **(B)** were shown, and adjusted odds ratio (aOR) with 95% confidence interval (CI) were adjusted for age, sex, comorbidities, clinical status, and site of infection as potential confounders. ^a^Fisher’s exact test was used to evaluate associations for “zero” frequency was present. ^b^Other drugs included tetracyclines and sulfamethoxazole-trimethoprim. BL-BLI, β-lactam/β-lactamase inhibitors (cefoperazone-sulbactam and piperacillin-tazobactam); KP, *Klebsiella pneumoniae*.

The 7- and 15-day treatment effectiveness were used as outcome indicators to analyze the effect of specific antimicrobial agents to reduce the impact of non-infectious factors on mortality. As shown in **Figure 5**, the use of ceftazidime-avibactam significantly improved the 7- and 15-day treatment effectiveness. The use of tigecycline also showed a statistical advantage in 7 days, while the use of polymyxin B did not exhibit a statistical advantage. The use of aminoglycosides and fosfomycin could improve the outcomes, possibly because of the sensitivity of CRKP to these drugs.

### Predictors of the High Cost of Antimicrobial Agents

Univariate and multiple analyses were conducted on data obtained from 140 patients whose survival time was >30 days to identify the predictors associated with the high cost of the antimicrobial agents (**Table 3**). High cost was defined as USD 4 300 (the median cost of antimicrobial agents of the surviving cohort). Male gender (odds ratio [OR], 3.73; 95% CI: 1.37–11.36; *P* = 0.014), high SOFA score (one-point increments, OR, 1.30; 95% CI: 1.08–1.61; *P* = 0.010), use of ceftazidime–avibactam (OR, 10.62; 95% CI: 1.70–207.22; *P* = 0.033), and use of tigecycline (OR, 8.37; 95% CI: 3.47–22.25; *P* < 0.001) were the independent predictors of the high cost of the antimicrobial agents. Our analysis on the cost of all medications also revealed that male gender, use of ceftazidime–avibactam, and use of tigecycline were the independent predictors (**Supplementary Table S1**).

**Table 3:** Factors associated with high cost of antimicrobial agents^a^.

Univariate analysis^b^	Low cost n = 70	High cost^c^ n = 70	*P* value	OR (95%CI)
Male sex, n (%)	46 (65.7)	59 (84.3)	0.013	
SOFA score, median (IQR)	3 (2, 3)	3 (3, 6)	0.002	
Primary bloodstream infection, n (%)	3 (4.3)	10 (14.3)	0.054	
Platelet, × 10^9^/L	260.23 ± 132.52	210.24 ± 130.93	0.030	
Antimicrobial regimens^d^, n (%)				
Carbapenems	27 (38.6)	37 (52.9)	0.091	
BL-BLI^e^	28 (40.0)	15 (21.4)	0.019	
Broad spectrum β-lactams	9 (12.9)	3 (4.3)	0.070	
Ceftazidime-avibactam	1 (1.4)	11 (15.7)	0.003	
Tigecycline	9 (12.9)	36 (51.4)	<0.001	
Polymyxin B	0 (0)	9 (12.9)	0.003	
Number of anti-KP drugs, median (IQR)	2 (1, 2.75)	2 (2, 3)	0.005	
Appropriate treatments in 3 days, n (%)	36 (51.4)	57 (81.4)	<0.001	
**Multiple logistic regression analysis**				
Male sex			0.014	3.73 (1.37 - 11.36)
SOFA, 1-point increments			0.010	1.30 (1.08 - 1.61)
Ceftazidime-avibactam			0.033	10.62 (1.70 - 207.22)
Tigecycline			<0.001	8.37 (3.47 - 22.25)

a Cost of antimicrobial agents of 30-day surviving patients during hospitalization were analyzed (USD1 = CNY6.5 in year 2021).

b Factors correlating with cost with P < 0.10.

c High cost was defined as ≥ USD 4 300; low cost was defined as < USD 4 300.

d Antimicrobial regimens referred to the antibiotics intended to be used against CRKP administered at the same time within 7 days after CRKP was detected.

e β-lactam/β-lactamase inhibitors (BL-BLI) included cefoperazone-sulbactam and piperacillin-tazobactam.

IQR, interquartile range; OR, odds ratio; CI, confidence interval; SOFA, Sequential Organ Failure Assessment; KP, Klebsiella pneumonia.

## Discussion

The nomogram is a simple and practical clinical system through which critically ill patients can be identified and provided with intensive medical intervention at the earliest to reduce mortality (Park, 2018). Based on multivariable Cox regression analyses, we established for the first time a mortality risk predictive nomogram for patients with CRKP infection. The variables selected were a respiratory failure (a serious condition that could lead to death) and SOFA score (a simple and valuable organ-injury score to assess the severity of the acute illness and to predict the mortality in critically ill patients) (Seymour et al., 2016; Raith et al., 2017). The nomogram also included procalcitonin, which is an established marker for severe systemic bacterial infection and sepsis (Wacker et al., 2013; Rhee, 2017). Older age, which is a well-known predictor of outcome, was also included (Rowe and McKoy, 2017). Furthermore, we included the administration of active therapy within 72 h of assessment as a predictive variable; the performance of the model improved substantially after the inclusion of this variable.

A prominent advantage of our nomogram is that all variables can be obtained through routine examinations. They should be assessed on the assessment day, which is the day on which the first positive microbiology report is obtained. Therefore, the nomogram helps identify the patients at high risk and in making decisions on the management. The C-index of the prognostic nomogram was 0.85, which displayed a high value for predicting fatal outcomes. The calibration plots exhibited fair concordance between the nomogram predictions and the actual observations for 7-, 15-, and 30-day overall survival. These results strongly support the usefulness of the nomogram in patients with CRKP infection.

Previous studies have established that combination therapy provides a significant survival benefit against CRKP infection (Qureshi et al., 2012; Tumbarello et al., 2012; Daikos et al., 2014). A few studies directly analyzed the benefits of three-drug combination therapy in clinical outcomes, and two recent systematic reviews of the literature concluded that there were no significant differences in the likelihood of mortality between those treated with 2- and ≥3-drug combination regimens (Agyeman et al., 2020a; Agyeman et al., 2020b). The nomogram-based risk stratification performed in our study suggested that the three-drug combination regimen was beneficial in improving the outcomes in high-risk patients. However, our findings did not support the use of a three-drug combination regimen as it did not seem to be more effective than monotherapy in the low-risk group. The cost analysis also signified that multidrug combinations significantly increased the cost of antimicrobial agents and total medications. Hence, nomogram-based clinical decisions resulted in not only survival gains but also financial gains.

Since mortality is also influenced by other factors such as underlying diseases (apart from infectious factors), we used treatment efficiency as an outcome indicator to assess the efficacy of antimicrobial drugs. The results showed that ceftazidime–avibactam, fosfomycin, and amikacin were effective against infections caused by CRKP, which is consistent with the survival analysis and the findings of our previous study (Yao et al., 2021). This observation suggests that in cases where CRKP is sensitive to these drugs, their use should be prioritized (Shirley, 2018; Erturk Sengel et al., 2020). However, in some cases, the detected strains were only sensitive to polymyxin B and tigecycline; hence, regimens based on these drugs could be considered (Rafailidis and Falagas, 2014; Tilahun et al., 2021; Tompkins and van Duin, 2021). Tigecycline is recommended as an option for the treatment of multiple drug-resistant bacterial infections; nonetheless, its efficacy remains controversial (Yahav et al., 2011; Ni et al., 2016; Xiao et al., 2018). The results of our study indicated that tigecycline improved treatment efficiency, but survival analysis showed an increase in mortality, albeit the difference was not statistically significant (HR, 1.69; 95% CI: 0.98–2.94; *P* = 0.061) and there was a trend of higher mortality rates after adjusting for the confounding factors (HR, 1.58; 95% CI: 0.84–2.98; *P* = 0.154). Polymyxin B did not show an advantage against CRKP, and the statistical power might be weak because there was only a relatively small proportion of polymyxin B used in our study population. Finally, cost analysis implied that the use of ceftazidime–avibactam or tigecycline exacerbates the cost of medications because of the high price of these drugs in China. Overall, the results suggested that CRKP infections, especially those caused by drug-resistant strains, continue to be a clinical therapeutic challenge, Therefore, there is a tremendous need for the development of effective and safe drugs.

Our study has some limitations. First, it was a single-center, retrospective study. Thus, several variables with potential effects might have influenced the results. However, multivariate analysis was performed to control for such confounders. Second, a small proportion of patients in our study received relatively new antibiotics such as ceftazidime–avibactam. Finally, molecular analysis for the confirmation of genes encoding carbapenemase production was not performed.

## Conclusion

In conclusion, the risk factors for 30-day mortality among adult patients with CRKP infection were established to be the presence of respiratory failure, high SOFA score, PCT > 5 ng/mL, age >65 years, and non-appropriate treatments in 3 days. The nomogram to predict mortality was built based on the risk factors, and it showed good performance in terms of calibration discrimination and clinical utility. Therefore, the nomogram can be used to identify high-risk patients with CRKP infection. Three-drug combination therapy was observed to be associated with lower mortality in the high-risk patients but not in the low-risk group. Furthermore, combination therapy significantly increased the cost of antimicrobial agents and total medications. Prospective studies should be performed in the future to validate the predictive value of this model.

## Data Availability Statement

The raw data supporting the conclusions of this article will be made available by the authors, without undue reservation.

## Ethics Statement

The studies involving human participants were reviewed and approved by the Ethics Committee of XinHua Hospital Affiliated with Shanghai Jiao Tong University School of Medicine (XHEC-D-2021-130). Written informed consent for participation was not required for this study in accordance with the national legislation and the institutional requirements.

## Author Contributions

HuijY, FC, JC contributed to the conception of the study. JC, YY performed the data analyses. HuimY, Fang Wang contributed to the data analyses. JC, HuijY wrote the manuscript. SB, LL helped perform the analysis with constructive discussions. All authors contributed to the article and approved the submitted version.

## Funding

This work was supported by National Natural Science Foundation of China 82003742 (to JC); the Shanghai “Rising Stars of Medical Talent” Youth Development Program -Youth Medical Talents - Clinical Pharmacist Program SHWSRS2020_087 (to HY).

## Conflict of Interest

The authors declare that the research was conducted in the absence of any commercial or financial relationships that could be construed as a potential conflict of interest.

## Publisher’s Note

All claims expressed in this article are solely those of the authors and do not necessarily represent those of their affiliated organizations, or those of the publisher, the editors and the reviewers. Any product that may be evaluated in this article, or claim that may be made by its manufacturer, is not guaranteed or endorsed by the publisher.

## References

[B1] Agyeman A. A. Bergen P. J. Rao G. G. Nation R. L. Landersdorfer C. B. (2020a). A Systematic Review and Meta-Analysis of Treatment Outcomes Following Antibiotic Therapy Among Patients With Carbapenem-Resistant *Klebsiella pneumoniae* Infections. Int. J. Antimicrob. Agents 55, 105833. doi: 10.1016/j.ijantimicag.2019.10.014 31730892

[B2] Agyeman A. A. Bergen P. J. Rao G. G. Nation R. L. Landersdorfer C. B. (2020b). Mortality, Clinical and Microbiological Response Following Antibiotic Therapy Among Patients With Carbapenem-Resistant *Klebsiella pneumoniae* Infections (A Meta-Analysis Dataset). Data Brief 28:104907. doi: 10.1016/j.dib.2019.104907 31886351 PMC6921139

[B3] Bengoechea J. A. Sa Pessoa J. (2019). *Klebsiella pneumoniae* Infection Biology: Living to Counteract Host Defences. FEMS Microbiol. Rev. 43, 123–144. doi: 10.1093/femsre/fuy043 30452654 PMC6435446

[B4] Brink A. J. (2019). Epidemiology of Carbapenem-Resistant Gram-Negative Infections Globally. Curr. Opin. Infect. Dis. 32, 609–616. doi: 10.1097/QCO.0000000000000608 31567571

[B5] Clinical and Laboratory Standards Institute (2021). M100: Performance Standards for Antimicrobial Susceptibility Testing. 31st ed (Wayne, PA, USA: Clinical and Laboratory Standard Institute). 10.1128/JCM.00213-21 PMC8601225 34550809

[B6] Daikos G. L. Tsaousi S. Tzouvelekis L. S. Anyfantis I. Psichogiou M. Argyropoulou A.. (2014). Carbapenemase-Producing *Klebsiella pneumoniae* Bloodstream Infections: Lowering Mortality by Antibiotic Combination Schemes and the Role of Carbapenems. Antimicrob. Agents Chemother. 58, 2322–2328. doi: 10.1128/AAC.02166-13 24514083 PMC4023796

[B7] Durante-Mangoni E. Andini R. Zampino R. (2019). Management of Carbapenem-Resistant Enterobacteriaceae Infections. Clin. Microbiol. Infect. 25, 943–950. doi: 10.1016/j.cmi.2019.04.013 31004767

[B8] Erturk Sengel B. Altinkanat Gelmez G. Soyletir G. Korten V. (2020). *In Vitro* Synergistic Activity of Fosfomycin in Combination With Meropenem, Amikacin and Colistin Against OXA-48 and/or NDM-Producing *Klebsiella pneumoniae* . J. Chemother. 32, 237–243. doi: 10.1080/1120009X.2020.1745501 32228228

[B9] Falcone M. Russo A. Iacovelli A. Restuccia G. Ceccarelli G. Giordano A.. (2016). Predictors of Outcome in ICU Patients With Septic Shock Caused by *Klebsiella pneumoniae* Carbapenemase-Producing *K. pneumoniae* . Clin. Microbiol. Infect. 22, 444–450. doi: 10.1016/j.cmi.2016.01.016 26850826

[B10] Munoz-Price L. S. Poirel L. Bonomo R. A. Schwaber M. J. Daikos G. L. Cormican M.. (2013). Clinical Epidemiology of the Global Expansion of *Klebsiella pneumoniae* Carbapenemases. Lancet Infect. Dis. 13, 785–796. doi: 10.1016/S1473-3099(13)70190-7 23969216 PMC4673667

[B11] Ni W. Han Y. Liu J. Wei C. Zhao J. Cui J.. (2016). Tigecycline Treatment for Carbapenem-Resistant Enterobacteriaceae Infections: A Systematic Review and Meta-Analysis. Medicine 95, e3126. doi: 10.1097/MD.0000000000003126 26986165 PMC4839946

[B12] Park S. Y. (2018). Nomogram: An Analogue Tool to Deliver Digital Knowledge. J. Thorac. Cardiovasc. Surg. 155, 1793. doi: 10.1016/j.jtcvs.2017.12.107 29370910

[B13] Pournaras S. Vrioni G. Neou E. Dendrinos J. Dimitroulia E. Poulou A.. (2011). Activity of Tigecycline Alone and in Combination With Colistin and Meropenem Against *Klebsiella pneumoniae* Carbapenemase (KPC)-Producing Enterobacteriaceae Strains by Time-Kill Assay. Int. J. Antimicrob. Agents 37, 244–247. doi: 10.1016/j.ijantimicag.2010.10.031 21236643

[B14] Qureshi Z. A. Paterson D. L. Potoski B. A. Kilayko M. C. Sandovsky G. Sordillo E.. (2012). Treatment Outcome of Bacteremia Due to KPC-Producing *Klebsiella pneumoniae*: Superiority of Combination Antimicrobial Regimens. Antimicrob. Agents Chemother. 56, 2108–2113. doi: 10.1128/AAC.06268-11 22252816 PMC3318350

[B15] Rafailidis P. I. Falagas M. E. (2014). Options for Treating Carbapenem-Resistant Enterobacteriaceae. Curr. Opin. Infect. Dis. 27, 479–483. doi: 10.1097/QCO.0000000000000109 25259809

[B16] Raith E. P. Udy A. A. Bailey M. McGloughlin S. MacIsaac C. Bellomo R.. (2017). Prognostic Accuracy of the SOFA Score, SIRS Criteria, and qSOFA Score for In-Hospital Mortality Among Adults With Suspected Infection Admitted to the Intensive Care Unit. JAMA 317, 290–300. doi: 10.1001/jama.2016.20328 28114553

[B17] Rhee C. (2017). Using Procalcitonin to Guide Antibiotic Therapy. Open Forum Infect. Dis. 4, ofw249. doi: 10.1093/ofid/ofw249 28480245 PMC5414114

[B18] Rodríguez-Medina N. Barrios-Camacho H. Duran-Bedolla J. Garza-Ramos U. (2019). *Klebsiella variicola*: An Emerging Pathogen in Humans. Emerg. Microbes Infect. 8, 973–988. doi: 10.1080/22221751.2019.1634981 31259664 PMC6609320

[B19] Rowe T. A. McKoy J. M. (2017). Sepsis in Older Adults. Infect. Dis. Clin. North Am. 31, 731–742. doi: 10.1016/j.idc.2017.07.010 29079157

[B20] Seymour C. W. Liu V. X. Iwashyna T. J. Brunkhorst F. M. Rea T. D. Scherag A.. (2016). Assessment of Clinical Criteria for Sepsis: For the Third International Consensus Definitions for Sepsis and Septic Shock (Sepsis-3). JAMA 315, 762–774. doi: 10.1001/jama.2016.0288 26903335 PMC5433435

[B21] Shirley M. (2018). Ceftazidime-Avibactam: A Review in the Treatment of Serious Gram-Negative Bacterial Infections. Drugs 78, 675–692. doi: 10.1007/s40265-018-0902-x 29671219

[B22] Steyerberg E. W. Vergouwe Y. (2014). Towards Better Clinical Prediction Models: Seven Steps for Development and An ABCD for Validation. Eur. Heart J. 35, 1925–1931. doi: 10.1093/eurheartj/ehu207 24898551 PMC4155437

[B23] Tilahun M. Kassa Y. Gedefie A. Ashagire M. (2021). Emerging Carbapenem-Resistant Enterobacteriaceae Infection, Its Epidemiology and Novel Treatment Options: A Review. Infect. Drug Resist. 14, 4363–4374. doi: 10.2147/IDR.S337611 34707380 PMC8544126

[B24] Tompkins K. van Duin D. (2021). Treatment for Carbapenem-Resistant Enterobacterales Infections: Recent Advances and Future Directions. Eur. J. Clin. Microbiol. Infect. Dis. 40, 2053–2068. doi: 10.1007/s10096-021-04296-1 34169446 PMC8527571

[B25] Tsioutis C. Eichel V. M. Mutters N. T. (2021). Transmission of *Klebsiella pneumoniae* Carbapenemase (KPC)-Producing *Klebsiella pneumoniae*: The Role of Infection Control. J. Antimicrob. Chemother. 76, i4–i11. doi: 10.1093/jac/dkaa492 33534880

[B26] Tumbarello M. Viale P. Viscoli C. Trecarichi E. M. Tumietto F. Marchese A.. (2012). Predictors of Mortality in Bloodstream Infections Caused by *Klebsiella pneumoniae* Carbapenemase-Producing *K. pneumoniae*: Importance of Combination Therapy. Clin. Infect. Dis. 55, 943–950. doi: 10.1093/cid/cis588 22752516

[B27] Wacker C. Prkno A. Brunkhorst F. M. Schlattmann P. (2013). Procalcitonin as A Diagnostic Marker for Sepsis: A Systematic Review and Meta-Analysis. Lancet Infect. Dis. 13, 426–435. doi: 10.1016/S1473-3099(12)70323-7 23375419

[B28] Xiao T. Yu W. Niu T. Huang C. Xiao Y. (2018). A Retrospective, Comparative Analysis of Risk Factors and Outcomes in Carbapenem-Susceptible and Carbapenem-Nonsusceptible Bloodstream Infections: Tigecycline Significantly Increases the Mortality. Infect. Drug Resist. 11, 595–606. doi: 10.2147/IDR.S153246 29731648 PMC5926074

[B29] Yahav D. Lador A. Paul M. Leibovici L. (2011). Efficacy and Safety of Tigecycline: A Systematic Review and Meta-Analysis. J. Antimicrob. Chemother. 66, 1963–1971. doi: 10.1093/jac/dkr242 21685488

[B30] Yao H. Liu J. Jiang X. Chen F. Lu X. Zhang J. (2021). Analysis of the Clinical Effect of Combined Drug Susceptibility to Guide Medication for Carbapenem-Resistant *Klebsiella pneumoniae* Patients Based on the Kirby–Bauer Disk Diffusion Method. Infect. Drug Resist. 14, 79–87. doi: 10.2147/IDR.S282386 33469322 PMC7812027

