# Supplementary material for: Prediction of Prognosis in Adult Patients With Carbapenem-Resistant Klebsiella pneumoniae Infection
Source: Front Cell Infect Microbiol. 2022 Jan 11;11:818308. doi: 10.3389/fcimb.2021.818308 (PMC8787092; doi:10.3389/fcimb.2021.818308)
Supplement: Supplementary file 1 [file DataSheet_1.docx]

Supplementary Material


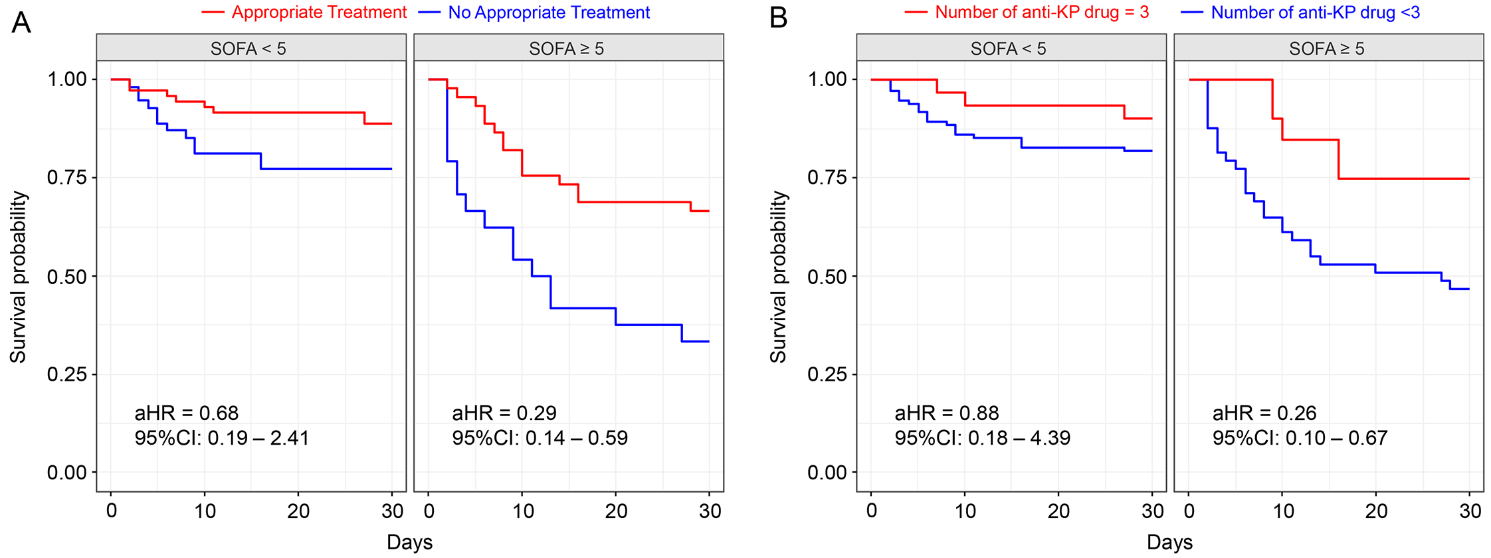


Supplementary Figure S1. Kaplan–Meier survival plots for different treatment regimens associated with Sequential Organ Failure Assessment (SOFA) stratification were shown, and adjusted hazard risk ratios (aHR) with 95% confidence intervals (CI) were examined using the Cox regression analysis adjusted for age, sex, comorbidities, clinical status, and site of infection as potential confounders.

**Supplemental Table S1.** Factors associated with high cost of medications^a^

| Univariate analysis^b^ | Low cost n = 70 | High cost^c^ n = 70 | *P* value | OR (95% CI) |
| --- | --- | --- | --- | --- |
| Male sex, n (%) | 47 (67.1) | 58 (82.9) | 0.034 |  |
| SOFA score, median (IQR) | 3 (2, 4) | 3 (3, 6) | 0.013 |  |
| Mechanical ventilation, n (%) | 47 (67.1) | 56 (80.0) | 0.087 |  |
| Gastric catheterization, n (%) | 56 (80.0) | 64 (91.4) | 0.060 |  |
| Platelet, × 10^9^/L | 256.20±133.81 | 214.27±131.05 | 0.067 |  |
| **Antimicrobial regimens^d^, n (%)** |  |  |  |  |
| Carbapenems | 26 (37.1) | 38 (54.3) | 0.043 |  |
| BL-BLI^e^ | 27 (38.6) | 16 (22.9) | 0.046 |  |
| Broad spectrum β-lactams | 9 (12.9) | 3 (4.3) | 0.070 |  |
| Ceftazidime-avibactam | 1 (1.4) | 11 (15.7) | 0.003 |  |
| Tigecycline | 14 (20.0) | 31 (44.3) | 0.003 |  |
| Polymyxin B | 0 (0) | 9 (12.9) | 0.003 |  |
| Number of anti-KP drugs, median (IQR) | 2 (1, 2) | 2 (2, 3) | 0.002 |  |
| Appropriate treatments in 3 days, n (%) | 40 (57.1) | 53 (75.7) | 0.021 |  |
| **Multiple logistic regression analysis** |  |  |  |  |
| Male sex |  |  | 0.045 | 2.43 (1.04 - 6.02) |
| Ceftazidime-avibactam |  |  | 0.012 | 15.38 (2.70 - 292.41) |
| Tigecycline |  |  | 0.003 | 3.29 (1.52 - 7.38) |

^a^Cost of medication of 30-day surviving patients during hospitalization were analyzed (USD1 = CNY6.5 in year 2021).

^b^Factors correlating with cost with *P* < 0.10

^c^High cost was defined as ≥ USD 11 000; low cost was defined as < USD 11 000.

^d^Antimicrobial regimens referred to the antibiotics intended to be used against CRKP administered at the same time within 7 days after CRKP was detected.

^e^β-lactam/β-lactamase inhibitors (BL-BLI) included cefoperazone-sulbactam and piperacillin-tazobactam.

Abbreviations: IQR, interquartile range; OR, odds ratio; CI, confidence interval; SOFA, Sequential Organ Failure Assessment; BL-BLI, β-lactam/β-lactamase inhibitors; KP, Klebsiella pneumoniae
